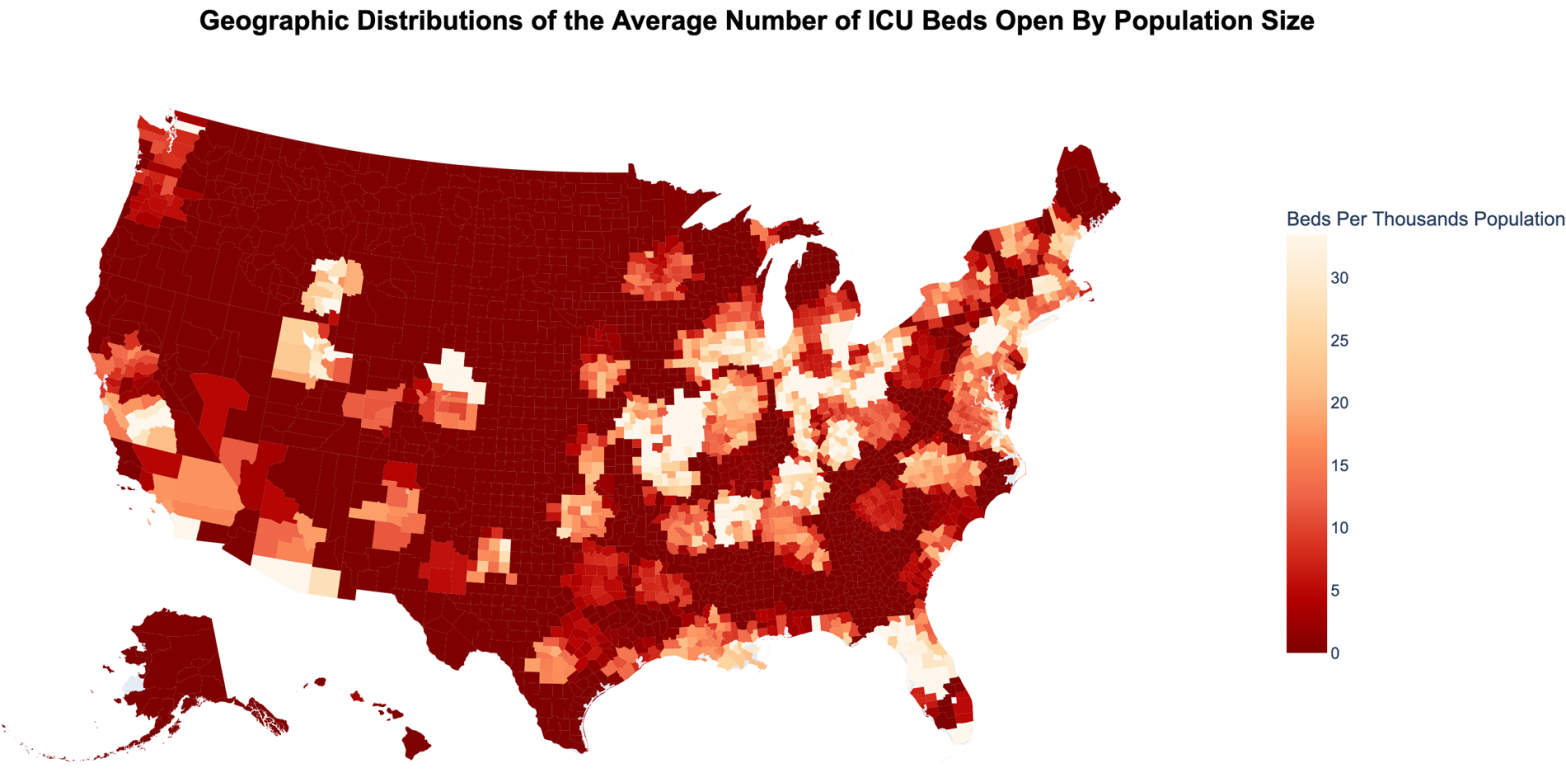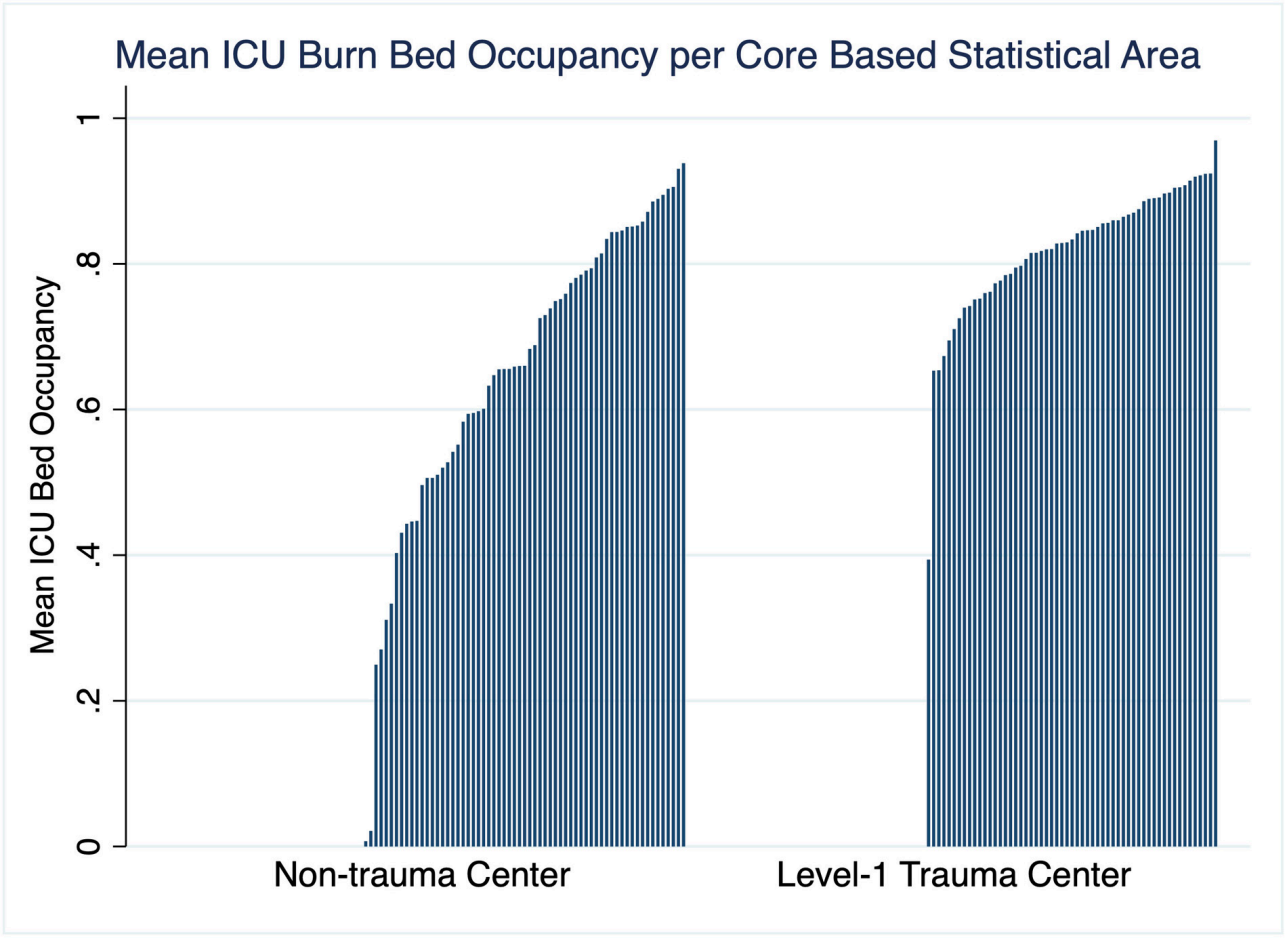# 101 US Capacity for Burn Care in a Mass Causality Incident

**DOI:** 10.1093/jbcr/iraf019.101

**Published:** 2025-04-01

**Authors:** Allen Green, Jeff Choi, Kristan Staudenmayer, Dong Hur, Andrew Ibrahim, Clifford Sheckter

**Affiliations:** Stanford University School of Medicine; Stanford University Department of Surgery; Stanford University; University of Michigan; University of Michigan; Stanford University

## Abstract

**Introduction:**

Preparedness for a Mass Causality Incident (MCI) involving burns is important to ensure national safety. Burn care is particularly vulnerable to becoming overwhelmed given the intensity of resources required to care for a severely injured burn patient juxtaposed to a limited number of burn centers in the US. We aimed to characterize capacity for severely injured burn patients across the US and simulate preparedness for an MCI across Core Base Statistical Areas (CBSA).

**Methods:**

The COVID-19 pandemic prompted the Department of Health and Human Service to record bed occupancy and capacity for all US hospitals on a weekly basis from January 1st, 2020 to May 1, 2024. Hospitals were grouped into their CBSA according to the Department of Housing and Urban Development. Hospitals were characterized based on burn center status (defined by Centers for Medicare & Medicaid Services Provider of Service file) and level-1 trauma center status (American College of Surgeons), given many mechanisms of burn-related MCI would require trauma care. MCI scenarios were considered with 10, 25, and 50 critically injured burn patients requiring intensive care unit (ICU) beds. Population level estimates for preparedness were based upon number of lives in each CBSA in 2023.

**Results:**

There were 150 burn centers across 104 CBSAs. Of CBSAs with burn centers, 57 (55%) had combined burn/trauma centers. Post-pandemic (2022 onward), median ICU bed occupancy at hospitals with burn centers was 81.0% (IQR 64.8%, 89.0%). Occupancy was higher if a burn center was also a level-1 trauma center (86.1% vs 72.3%, p< 0.001). Post-pandemic by CBSA, the median number of open ICU beds per million residents at hospitals with burn centers was 14.0 (IQR 4.6, 36.3); at combined burn/trauma centers it was 15.4 (IQR 4.9, 40.0). 29% of CBSAs did not have sufficient ICU beds to manage >10 severely burned patients. 75% of CBSAs did not have sufficient ICU beds to manage >25 or more severely burn patients. Only 4% of CBSA had enough open ICU beds to manage 50 severely burned patients. Per capita, the bottom quartile of CBSAs for open ICU beds at hospitals with burn centers represented 35% of the entire US population, indicating constrained capacity in regions at high risk for MCI.

**Conclusions:**

45% of CBSAs with burn centers were not equipped to care for polytrauma burn patients. The most vulnerable regions of the US for a burn MCI were also the more densely populated.

**Applicability of Research to Practice:**

These real-world data show a mismatch between need and capacity of burn and combined burn/trauma ICU beds across the US in event of a burn MCI.

**Funding for the Study:**

N/A